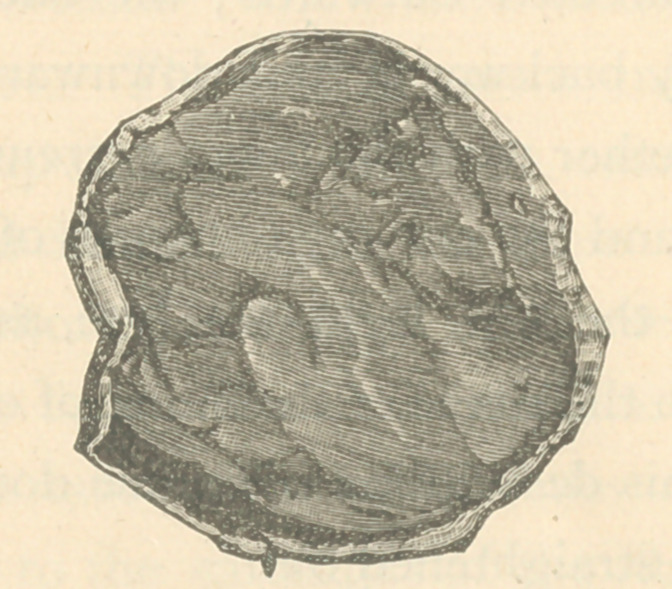# Mollities Ossium

**Published:** 1885-05

**Authors:** W. J. Webb

**Affiliations:** 728 Lincoln Avenue; Chicago


ARTICLE II.

A CASE MOLLITIES OSSIUM. *By* W. J. WEBB, M. D., *of Chicago.*

[Read before the Chicago Medical Society, March 2nd, 1885.]

The etiology and pathology of this disease is very obscure and its clinical history meager and contradictory. So far, the most positive information we have upon the affection has come to us from Europe, the valleys of the Rhine and Po seeming to furnish the most examples. Yet the knowledge thus obtained is so varied, and the reports so contradictory, that it is far from being conclusive or satisfactory. That it often bears some relation to the pregnant state, though, is probable. Debossati, of Milan, claims to have treated no less than sixty-two cases, for deformities of the pelvis, caused by malacosteon, during a period of eighteen years. They were all women who came from districts where pellagra and typhus were most prevalent. This statement is strengthened by the collection of the records of one hundred and forty-five cases by Mr. Durham, who says that of this number one hundred and thirty-two were women, and that ninety-one of them were attacked during pregnancy or shortly after childbirth, and that the majority of them were between twenty-five and thirty-five years of age. This corresponds somewhat also with two cases reported by Dr. Bush in the *London Practitioner*, 1883. As has just been intimated, the social habits and character of the food may play an important part in the predisposing causes. The sixty-two cases referred to by Debosati came from wretched villages, where their food consisted mostly of maize or rice, and that often musty. The rooms in which they worked were filthy and badly ventilated. These unfortunate surroundings and circumstances, added to the character of the water, and the meager subsistence with which they eke out an existence,

Debossati supposes to be largely concerned in the production of the disease.

Agnew says that rickets, struma syphilis, miasmatic emmenations, rheumatism, dampness and food deficient in certain universal substances have all been invoked in explanation of the morbid change. In some few instances, as in one given by Dr. Omerod (in the *Medical Record*, in Sept., '57), the disease seems to be hereditary. Mental disease, aliment poor in saline constituents of bone, have also been mentioned as exercising a determining influence. The testimony in reference to the condition of the renal secretion in this malady, while it has brought us a step nearer in our investigation, has been so limited and contradictory, that nothing very satisfactory has been obtained from this source. That this secretion has usually presented marked alterations in quality and appearance, is generally, I think, admitted; yet there are some few cases, we are told, in which no apparent deviation whatever from the healthy constitution of the urine is observed during the course of the disease. The older writers described to us a copious deposit of a white, chalky-like substance that was found upon evaporation or cooling; this, later and more thorough investigations have shown to consist mainly of phosphate of lime. Now, while this discovery would at first thought seem to furnish us a key to the solution of this abnormal metamorphosis, I think that upon more deliberate consideration it will be seen that we are nearly as far from the real cause as we were before the revelation was made. Being convinced that these lime salts have been removed from their proper relations in the bones and then finding them in their disintegrated state on the glass slide or in the test tube, only convinces us that they have been eliminated and indicates to us the means by which it was done, but it does not in any way intimate to us with any degree of certainty the cause or causes that were instrumental in originating and perpetuating this destructive process. Some pathologists tell us that the earthy matter in the hyaline matrix is first dissolved, thus reducing the compact bony structure to a condition akin to the original cartilage; that as the disease advances this substance is disorganized or dissolved, and these heterogeneous masses that have been variously described as fatty, jelly-like products, are the result of these changes. The venerable John Hunter considered these changes simply a fatty degeneration and Paget seems to give credit to the same opinion, saying that the pink color of the medullary substance is due to the bright tints of a part of the oil globules and of the nuclei and granules in the bright-colored fat cells; and that there was no appearance of an excess of blood in the bone or any part of its contents. Holmes says, quoting from the *Medico-Chirur. Transactions*, that the diseased tissues consisted simply of an organic matrix, with a little earthy matter, and containing a little fat; hence it is impossible to describe the disease as simply fatty degeneration. But the authorities who have had most to say upon the pathology of the disease, and whose professional standing and scientific research render their opinions worthy of our careful consideration, are Virchow and Rindfleisch. And still they do not agree; Virchow claiming that the first stages of the abnormal vital processes are essentially that of hyperæmia, and that the medullary substance is reduced to a condition identical with the fœtal marrow, and that it is caused by the presence of lactic acid in the parts, while Rindfleisch says the condition is not one of congestion or hyperæmia, but an extravasation of blood in the part, and that the medullary substance resembles more nearly the tissue of normal spleen, and that the disintegration is caused by the presence of carbonic acid in the parts.

McNamara says: "We can, however, form only an imperfect notion of the pathology of the disease until we examine sections through the extremities of the bones which are still comparatively healthy; here we find the articular cartilages in places unaffected, the ossification is progressing in a very perfect and orderly manner, but the cancellated tissue a short distance from the cartilage is filled with round cells; in other places composed of a fine fibro-cellular material similar to that of cartilage when it has undergone fibroid degeneration and broken down into a cavity or ulcer. In some sections we see the cells of the articular cartilages undergoing very peculiar changes, each of the original cells breaking up into a nest of cells, which individually resemble the blood globules of a fœtus. In fact, it seems as though the early stages of the disease existing in these specimens are nearly related to the condition noticed in cases of lympho-sarcoma: the cells of the medulla, from some obscure cause which we do not understand, have failed to perform their functions, their tendency under the circumstances being to revert to their cartilaginous prototype, as though there were something defective in the living matter of the blood. Presuming that the sound cells of the medulla are organisms, the outcome of the cartilage and blood cells in the disease, we are considering, the living matters of the blood being defective, the inherited tendency of the cartilage cells predominate, and so the medulla, in place of building up new bone to replace that which disappears in the course of disintegration, breaks up into a lowly organized fibroid structure and subsequently degenerates into a fatty substance such as that which we see filling the cancellated structure of the bones."

This excellent and ingenious investigator of this subject concludes that the cases of osteomalacia may be divided into three classes:—

First. Those due to carcinomatous or sarcomatous disease of the medulla.

Second. Those due to an affection of the medulla allied to lymphoma.

Third. Those due to some peculiar condition of the medulla or perhaps of the blood, dependent on the state of pregnancy.

This, to my mind, covers the ground as intelligently and completely as anything I have seen; yet it seems to me that there must be a condition of the nutritive fluid not connected with pregnancy that predisposes to these various changes in the medullary cavity. Whether it is lactic acid or carbonic acid, or both, or something else, I am not prepared to say. The variety of forms in which the disease manifests itself and the various pathological conditions that have already been discovered would indicate that it is often complicated and the diseased processes far from being uniform; and I am inclined to the opinion that in the future investigations of the disease the functions of the nervous system will receive more attention than formerly.

As to the cause of the case, I will endeavor to describe to you, I have no well grounded conviction; but since he dates his symptoms back of the specific disease, and since some of the other members of the family have similar symptoms, it seems to me reasonable to infer that in the predisposing causes an hereditary tendency may have existed, and this, together with his youthful vice, coupled with his spermatorrhœa and erotic temperament, may have been sufficient to induce the albuminuria and a consequent cachexia, which served as a fruitful soil for the ravages of the specific disease, which I think he had in 1876.

The following is a clinical history of a case of this disease which came under my observation last year, with plates illustrating the external appearances of the body and a part of some of the bones. I have to regret that circumstances forbade my obtaining microscopical examinations of the tissues and secretions.

I was called to visit Mr. G. about the 1st of August, 1884. I found him lying upon his back, and barring his somewhat labored breathing, to all external appearances comparatively comfortable, and judging from the looks of his face one would say that he was well nourished; but upon removing the covering from his body I found it presented quite a different aspect. His right arm was bent and broken, as were his femora. The anterior part of the chest wall was directed forwards and upwards, and by placing my hand upon it I could distinctly feel what to the sense of touch reminded one of a counting abacus under the skin, so nodulated were his ribs.

At this visit my attention was directed to the most peculiar effects of the disease I noticed during the time he was under my observation. It consisted in what seemed to be the separation of the left supraorbital ridge. He said it was loose, and taking hold of it I found it was detached from the continuous bony structure, and when it was moved the patient said he could hear a grating noise. It soon afterwards partially united, as did many of the other fractures, and remained so until death. The maxillary bones at that time, as were the teeth, were quite firm and unyielding. The joints were generally slightly enlarged and irregular in outline. His appetite good. Temperature, 97½° F., circulation, 99, with what I considered an aortic heart murmur. Bowels constipated, tongue slightly coated with a light yellow fur, respiration 22. Urine, in twenty-four hours, 136 fl. ozs; acid, reaction; specific gravity, 1,021, with heavy deposit of phosphates; a trace of albumen, but no sugar. Microscopical examination was not made. The patient seemed to be a little cyanosed and jaundiced, with some spehorrhœa, especially of the scalp and hairy parts of the body, accompanied also with severe itching of various parts at times, which, I think, was due largely to want of proper bathing, I noticed also at this time evidences of specific disease. On the front of the leg there were such characteristic spots and cicatrices as to be unmistakable, as there were also on the arms, shoulders and upper part of the chest. Yet he unequivocally asserted his innocence till the last. His hair was dark and quite normal in appearance ; his beard was comparatively thin and confined chiefly to the chin and upper lip. The genital organs were diminutive in size and retracted. On Oct. 6, 1884, I photographed the body, and noted the following history as given to me by the patient. He was born in Lycoming county, Pa., in 1848, and was brought up as a laborer, being engaged in the woods, sawmills and on the farm. He was of German descent, but his parents were born in this country. His father is living, aged seventy-four years. Health always poor; suffers from urinary trouble. His mother died at the age of forty-six years—cause unknown. His father's father at sixty years of age, father's mother at nineteen years of age. Mother's father at eighty years of age, mother's mother at fifty-four years of age. Has one brother, aged thirty-one years. Health poor. Has heart and lung disease. Has five sisters, aged as follows: Oldest, forty-five, health not good, has liver difficulty, cannot work much; second, aged forty-three years, health also poor, and is mentally incompetent to perform the duties of life—seems to suffer from melancholia, subjective symptoms much the same as patient's, severe aching in bones. The remaining three aged respectively thirty-nine, thirty-four and thirty, are all living, with rather poor health. About the only vice the patient acknowledged to have indulged in, is that of onanism, which he commenced at the early age of eight years and continued moderately during his youth, but says that he was always very passionate in this particular, so much so that it seemed almost impossible for him to satisfy this appetite after he was married, and that this morbid sensation did not forsake him until a few weeks before I saw him. Said, also, that he often had quite profuse discharges of a milky-looking substance from the penis before or after voiding his urine. This has troubled him at irregular intervals since his youth. At nine years of age he claims to have had a sunstroke, which was probably a convulsion of some kind. Previous to this time he always suffered from indigestion and diarrhœa, during which he passed much undigested food, after which [the sunstroke] he was always constipated. Since his sixteenth year, he has been tired and sleepy, would at times even lie down and go to sleep in the midst of his work; and that heavy lifting always caused pain in his bones. He always had a ravenous appetite, that was hardly ever satisfied, and drank a great deal of water, coffee and tea, but no alcoholic stimulants. In 1870 or '71, he went to Nevada to work in the woods. There he suffered considerably from catarrh and dryness of the throat. Ate a good deal, and had fits after meals. Remained three months and went to California, where his health seemed to be better, but there his appetite was poor. In 1873 went back to Nevada to work in the mines. There he drank much water that was sweet and it was said contained large quantities of arsenic. At this place he first noticed the eruption on the skin, which he was told was caused by the arsenic. At the same time he had sore throat, and his hair came out. He said, also, that many of the miners suffered as he did, but not so severely. All had more or less of the eruption. After ten months, he was taken away by an uncle, being at the time badly bloated and unable to work. Next day after he was removed he had a severe attack of dysentery, which lasted two weeks, from the effects of which he thinks he never recovered.

In 1875, went back to chopping again, but was obliged to desist, as he could not stand it, having such severe pains in his stomach and bones. Had, also, at this time a very bad taste in his mouth, especially in the morning. Here the eruption was much worse; often it would assume the character of blood boils. During this time he had a tape-worm removed. In 1876, had a severe struggle with a vicious horse, and suddenly felt sharp, shooting pains in various parts of the body; shortly afterwards became very sore all over, had severe deep-seated pains in various places, especially in the arms and thighs. This condition continued with more or less regularity and severity until the summer of 1882, when he gave up work and took to his crutches. In 1877, he was married and has two apparently healthy children, aged two and four years.

His wife appears healthy, but prematurely old. Had another tape-worm removed in 1880. Had his urine examined in 1875 and 1883. In 1875, it was of light specific gravity, and contained albumen. In '83, he passed twelve pounds in twenty-four hours. It also contained albumen. Two weeks before he died, I made a chemical analysis of his urine and found it contained a peculiar albuminoid substance, which would coagulate upon the addition of nitric acid, but would again redissolve by the application of heat. This, I suppose, was the substance described by Dr. Bence Jones and others as the deutoxide of albumen.

During this long history he was treated by many physicians for many diseases, and by those professing as many different faiths. Being once badly salivated, since which time he has not had much to do with the so-called old school doctors. Hence I did not dare mention medicine to him for a long time, but simply allowed him to take his acid phosphates, which he thought was doing him much good. He went to bed finally in January, 1884. Measurement of the body on October 6, 1884: Lower right extremity from symphysis pubis to internal malleolus, 23 inches; to the left internal malleolus, 26 inches; across the femora, just below the great trochanters, 20 inches; right tibia, 16 inches; left, 17½; sternum, 7 inches. The vault of the cranium could be quite easily depressed, and seemed flexible. Total length of body, 52 inches; original length, 68 inches; pulse, normal; respiration 23,—labored; some ascites; appetite good; sleeps well; mind clear and memory good; expresses himself well. October 13, tongue slightly coated, pulse, 102; temperature, 99.10° F.; respiration, 23; appetite good; rests well; mind clear; arms both comparatively useless. After my first visit his right humerus had united sufficiently to enable him to use it in fanning himself, but soon afterwards the bones in both arms became soft, and the upper extremities entirely useless. Thorax continues to flatten, and the organs of respiration and circulation more embarrassed. October 20, condition much the same. October 30, temperature 97½° F.; pulse 94, quite regular, but weak and soft; breathing more labored, and patient considerably cyanosed. Had a bad spell on the day before, during which he became very black (as he said) and thought he was going to die. Heart's action exaggerated, with the first sound increased while the second is scarcely audible. November 10, unable to move any member of the body except the right hand slightly, and the fingers and toes a little. Lower extremities completely everted, and contained so much fluid that they would pit considerably upon pressure. Temperature 99° F.; pulse, 112; respiration, 24; vomits regularly every day, but appetite good; sleep much disturbed by dreams. November 12, the vital powers seem to be giving away. Pulse, 120; temperature, 99.9° F.; respiration, 25. November 13, much the same. November 20, considerably improved. Pulse, 90; temperature, 97½° F.; respiration less labored and 22 per minute. During the last few days has confined his diet to raw beef steak, which he says agrees with him. He cannot digest anything else as well; even milk causes distress and vomiting. January 7, 1885. Has yet more difficulty in breathing; thinks he is choking to death. Thorax very much flattened and the ribs scarcely perceptible in many places. Complains of considerable pains in upper part of right lung, which is nearly one-third above the clavicle. Temperature not taken; pulse, 115; respiration, 28, and performed with the utmost difficulty; obstinately constipated; some eruption on the face resembling pustular acne, and one small ulcer on the tongue. All the copper-colored spots disappeared from the body several weeks before. He coughs some and raises considerable viscid mucus. After the last named date he continued to fail gradually with all the former symptoms more or less intensified, until January 19, when he died from exhaustion, caused by pressure upon the vital organs. Post mortem next day in the presence of Drs. Sieber, Porter and Gobs, together with a friend and relative of the patient and one or two others.

POST MORTEM APPEARANCES.—His body was much flattened and diminished in size, so that its general contour resembled much that of a common frog. Yet he was six or seven inches longer than before death, on account of the straightening and stretching of the body in laying it out. He was much emaciated, and the skeleton distorted in various ways. Entire length after the stretching, 4 feet 7 inches. Left femur, 11 inches; right, 11¾; left tibia, 14 inches; right, 13¼; sternum, 6½ inches; left humerus, 11 inches; right, the same; left ulna, 9 inches; right, 10 inches; diaphragm on the right side extends up to the fourth rib. On the left, about 1½ inches lower, but we must not forget the fact that the whole chest wall was directed upwards about two inches, possibly more. Heart in the median line and moderately distended with blood. The pericardium extended up to the second rib, and contains about one ounce of fluid. The structure of the lungs were quite normal in appearance, except the upper lobe of the right, which was bound down by recent pleuritic adhesion. In position, however they were much crowded upwards, a considerable of which was found above the clavicle. The liver was somewhat smaller than normal and slightly congested. The vena cava and pulmonary veins were filled with blood. The spleen and pancreas about half the normal size, very pale, and the tissue tougher than usual. The kidneys were slightly enlarged, irregular in outline, modulated, the capule thickened, the pyramids enlarged and the tubules partially filled with what seemed to be a calcarious deposit.

The pelvis of the kidney contained more fat than common. The bladder was moderately distended and about filled the pelvis. The peritoneum as well as the intestines and stomach presented an unusually pale and yellowish color. The bowels contained nothing but some hardened feces in the descending colon. The mesenteric glands were enlarged. The brain and spinal cord were not exposed; the head, however, was much smaller than formerly, as the hat he wore was much too large for him, the circumference of the hat on the inside being one and one-fourth inches more than that of the head. The osseous system was all in an abnormal condition; the vault of the cranium being as easily depressed as a moderately distended foot-ball, and nearly as flexible. The bones of the face, though, suffered least, which fact corresponds with most or all other reported cases, yet these were lacking in stability, as was evinced by their being tender to the touch before death, and the looseness of the teeth. The enamel of the teeth was unaffected by the disease. The clavicles were enlarged, elongated and much curved upwards and forwards, caused, no doubt, by the movements and direction given to them in the process of respiration. The thorax, as I have said, resembled that of a common frog more than that of a man. The sternum was not so much affected as most of the other bones, owing, I think, to its close proximity to the cartilages of the ribs, but it was not in its proper shape or position, the manubrium, together with the sternal ends of the clavicles extended upwards and forwards while the gladiolus pointed upwards and slightly backwards, making a decided curve posteriorly in that bone. In the anterior third of the shaft of the ribs nearly, or quite all, the proper bone structure was wanting, and the periosteum bent or folded upon itself, the anterior part riding back over the posterior, while the whole anterior aspect of the thorax was directed backwards and upwards, causing the so-called frog-shaped chest. This condition caused so much pressure upon the organs within that the breathing was largely diaphragmatic during the time I knew the patient, and the efforts in respiration were so exaggerated that certain of the laity who saw him called him the bellows. The spinal column, while its natural curves were increased, was enlarged, with a quite sharp and extended lateral curvature to the right in the dorsal region and a slight one to the left in the lumbar region. In the pelvis the distortion was as great as that in the upper part of the body. The *ossa innominata* were nearly at right angles with the horizontal plane of the pelvis, the *ilia* being crowded inwards, while the *ischia* were directed outwards; the sacrum forwards and the pelvis slightly backwards and downwards. This position of the pelvis, together with the lower extremities, including the everted position and the curving outwards of the femora served to remind one of the old Egyptian lyre, as the outline thus formed was much the shape of the frame of one of these ancient instruments. This description of course does not apply to the body after it was straightened out.

The thigh bones were about eleven inches long, and their general outline has been sufficiently indicated. The upper extremities at the autopsy presented nothing of special importance except they were very small and so soft and flexible that they could easily have been tied in a knot. The only specimens of bone removed from the body were the upper left of the right femur, with a part of the acetabulum, the sternum and a part of one rib. At the section of the femur only a trace of bony substance was seen adhering to the inner surface of the periosteum, but as we approached the head of the bone the cancellated structure became more characteristic, yet no where was so hard that it could not have been easily incised with an ordinary scalpel. When the periosteum was opened there run out quite an abundance of a sero-sanguineous fluid that completely enveloped, and seemed to permeate the liver-like substance that had taken the place of the medullary canal, and most all of the compact bone of the shaft. In the trochanters there was an augmentation of the soft cancellated bony substance containing a large deposit of fat. The neck seemed to be shortened and the great trochanters extended above the articulation with the acetabulum.

In reference to treatment, I have already intimated that I did not attempt anything special in that direction. First, because

I had no faith in drugs to do him any particular good in the stage in which I had found him. Secondly, I do not believe I could have induced him, during the first two months that I knew him, to take medicine in sufficient quantities to produce any perceptible effect. Of his own will he took Horsford's acid phosphates a part of the time, and an infusion of senna occasionally, to move his bowels. I did induce him after awhile to try for a few days iodide of potas., syr. of sarsaparil., as I did pyrophosphate of iron and quin., but he concluded they did him more harm than good, so would not take them but a day or two. There is a pill, however, know as the Wagner, that I believe has been used with apparent good results, yet the only report of their use I have seen is the two cases recorded in the *London Practitioner*, in 1883. The formula is as follows: R. Phosphori gr. 1/3—ss., syr. simplicis ʒii. Mix well and add pulv. glycyrrhiza ℥iiss., pulv. gummi arab. ℈iv., pulv. gummi tragacanth ℈ii., m. fl. pil. No. 250. S. One twice a day and increase. With these a Dr. Bush claims to have cured two cases. The first, a woman thirty years of age, attacked a few weeks after childbirth; the pelvis was much distorted, she was unable to walk, and with the use of these pills, together with rest in horizontal position, she, after five months, was able to go up and down stairs without difficulty.

The second case, a woman aged fifty, was reduced almost to a skeleton and greatly deformed; thorax much affected, femora bowed and fractured. Treated in bed seven weeks, and took Wagner's pills. At the end of one and a half years bones firm and patient could walk without pains or discomfort. The deformity remained.

From my observations of this case I believe that wherever practicable a water bed, some ingenious method of effecting moderate extension together with a properly adjusted cast to the chest might be of benefit to the sufferer. It would, I think, if applied early, have a tendency to preserve the natural shape of the thorax and counteract the contraction of the muscles upon which depend the various distortions of the skeleton and favorably modify the irritation of the sensitive tissues.

728 Lincoln Avenue.

## Figures and Tables

**Figure f1:**
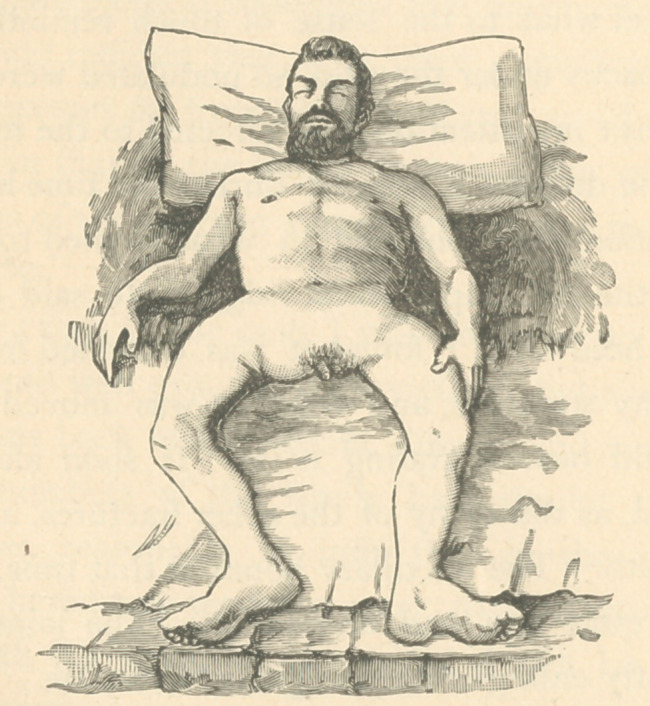


**Figure f2:**
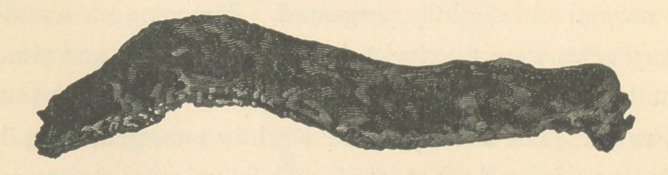


**Figure f3:**
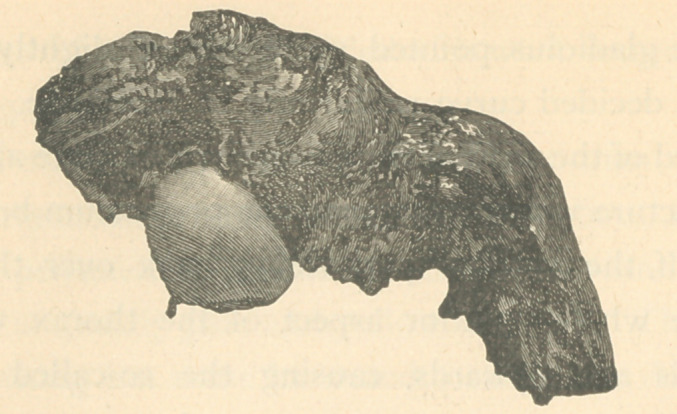


**Figure f4:**